# Two-Years Follow-Up of Symptoms and Return to Work in Complex Post-COVID-19 Patients

**DOI:** 10.3390/jcm12030741

**Published:** 2023-01-17

**Authors:** Erika Van Wambeke, Cécile Bezler, Anne-Marie Kasprowicz, Anne-Laure Charles, Emmanuel Andres, Bernard Geny

**Affiliations:** 1 Physiology and Functional Exploration Service, University Hospital of Strasbourg, NHC, 1 Place de l’Hôpital, CEDEX, 67091 Strasbourg, France; 2 Team 3072 “Mitochondria, Oxidative Stress and Muscle Protection”, Translational Medicine Federation of Strasbourg (FMTS), Faculty of Medicine, University of Strasbourg, 11 Rue Humann, 67000 Strasbourg, France; 3 Department of Internal Medicine, University Hospital of Strasbourg, 1 Place de l’Hôpital, CEDEX, 67091 Strasbourg, France

**Keywords:** long-COVID, post-COVID-19 condition, SARS-CoV-2, coordination team, prolonged symptoms, rehabilitation, employment

## Abstract

Introduction: Many COVID-19 patients present with severe long-lasting symptoms. They might benefit from a coordination team to manage such complex situations, but late efficacy still needs to be determined. Population and Methods: Out of 105 contacts, 45 patients had two phone consultations separated by personalized support 15 and 22 months, respectively, after COVID infection. Self-reported symptoms, feelings of improvement and ability to return to work allowed us to determine the efficacy of the therapeutic strategy proposed. Results: Unlike what was expected, many post-COVID-19 patients directly contacted the coordination team and had significant pre-existing comorbidities. Despite exercise, respiratory, olfactory rehabilitations, cognition/speech therapy and/or psychological support, the more frequent self-reported symptoms (fatigue, neurocognitive disorders, muscles and joint pain) did not resolve. However, dyspnea, anxiety and chest pain were significantly reduced. Finally, 2/3 of the patients felt some degree of improvement and returned to work either partially or fully, but 1/3 remained complaining of symptoms and out of work as late as 22 months after COVID occurrence. All patients greatly appreciated the second phone consultation. Conclusions: In such complex situations, besides early and adapted rehabilitations and psychological help allowing better symptom management, relatively simple actions such as a phone call might be very useful to reduce patients’ feelings of abandonment.

## 1. Introduction

As the SARS-CoV-2 pandemic continues to unfold around the world, many patients suffer from prolonged symptoms that might greatly affect their life. The definition of long or post-COVID-19 condition currently includes three important elements. First, an initial symptomatic episode of COVID-19 confirmed (PCR SARS-CoV-2 +, antigenic test SARS-CoV-2 + Serology SARS-CoV-2 +, anosmia/ageusia, bilateral pneumonia in frosted glass, etc.) or probable by the combination of at least three criteria of sudden onset in an epidemic context (fever, headache, fatigue, myalgia, dyspnea, cough, chest pain, diarrhea). Second, initial and prolonged symptoms not explained by another diagnosis not thought to be related to COVID-19, and third, symptom (s) lasting more than 3 months after infection and for at least 2 months after the acute phase of the disease [1]. This definition is very important, particularly the fact that symptoms cannot be explained by other diagnosis. Indeed, many patients suffering from post-COVID-19 condition might also demonstrate several and potentially serious comorbidities, making the link between symptoms and COVID infection difficult to discern.

Although still debated, despite beneficial effects of rehabilitation, the number of post-COVID patients might be high; thus, approximately 10% of COVID-19 patients may present with long-lasting symptoms, some of them being very disabling. More than a hundred different symptoms characterized by fluctuations and/or changes over time can result in poor quality of life and unemployment. After hospitalization, long-lasting symptoms have been related to previous comorbidities and intensive care unit length of stay [2,3,4,5,6,7,8,9], but this might be less clear in non-hospitalized patients. 

**Figure 1:**
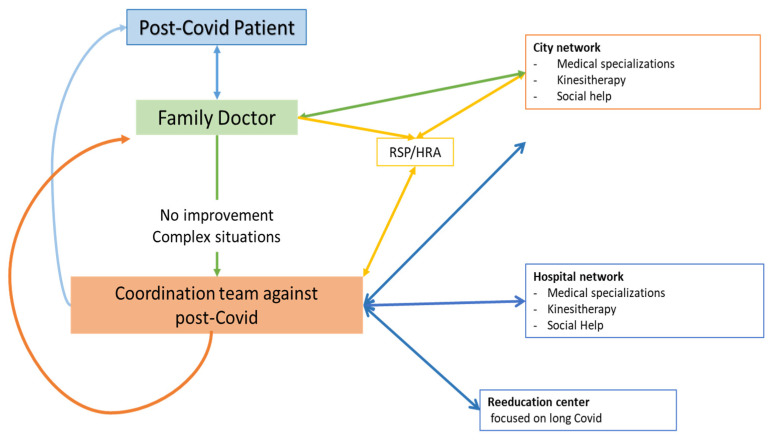
Structuration of post-COVID patients’ support. RSP: Regional Support Platform. HRA: Health Regional Agency.

At present, despite being hospitalized for COVID or non-hospitalized, patients with minimal or moderate severity of COVID infection regularly report their feelings of inadequate support, and their management remains challenging and controversial [10,11]. An adaptation of the health system to care for post-COVID patients therefore appears mandatory and in France, the attending physician is the cornerstone of management for patients with post-COVID-19 condition. As presented below (Figure 1), in most cases of post-COVID-19 condition, the family doctor directly manages the patients with the help of her/his city network. In the face of complex situations, including no improvement, the physician can obtain support from a coordination team, which will facilitate synthesis and access to all paramedical and medical facilities (city and hospital networks, rehabilitation centers). Such an organization, besides improving patients’ care, aimed also to avoid, at the systemic level, the drowning of the healthcare system both in the city and in hospital structures.

The aim of this prospective study was, therefore, to perform a long-term follow-up of symptoms and return to work of non-hospitalized post-COVID patients stuck in complex situations. 

## 2. Population and Methods

### 2.1. Population and Study Design

All queries addressed to the coordination team between September 2021 and March 2022 and either arising from the attending doctor, patients themselves or other structures were considered.

After this first contact, if needed, patients received a first phone consultation with an advanced practice nurse specialized in post-COVID-19 condition. The parameters collected for each patient included their medical history, demographic and clinical characteristics (age, gender, BMI, cardiovascular and other risk factors, etc.) and symptom descriptions. All patients were interviewed identically during the first and second phone call using a formalized list of symptoms. The question was whether patients had a particular symptom (yes or no). If the answer was yes, we asked patients to rate it from 1 to 10 (10 being the worst). These symptoms referred to “general health” and “mental state” and included fatigue, dyspnea, chest pain, muscle and/or joint pain, anosmia–dysgeusia, neurological disorders (headache, dizziness, difficulty concentrating or memory), digestive disorders, feelings of anxiety or depression. Subsequently, we asked patients whether or not they felt an improvement and if yes, to grade it as minimal, moderate or complete recovery.

During this 45 to 65 min consultation, we also obtained all previous medical consultations and investigations related to COVID, allowing us to complete a synthesis file. Based on these data, therapeutic proposals were focused on patients’ main symptoms.

Thereafter, a systematic second phone consultation was performed, particularly comparing patients’ symptom evolution and whether they returned to work.

The study was approved by the ethics committee of the University of Strasbourg (CE-2022-108, 12/10/2022) and all participants orally gave their informed consent.

### 2.2. Statistical Analysis

Data were analyzed with GraphPad Prism 8 software (GraphPad Software, Inc., San Diego, CA, USA). Nominal data were compared using two-tailed chi-squared tests and are presented as numbers/percentages. We used McNemar’s chi-squared test for matched pairs analysis. Quantitative values were expressed as mean ± SD. Statistical significance was considered at *p* < 0.05.

## 3. Results

### 3.1. Flow Chart of the Study

**Figure 2:**
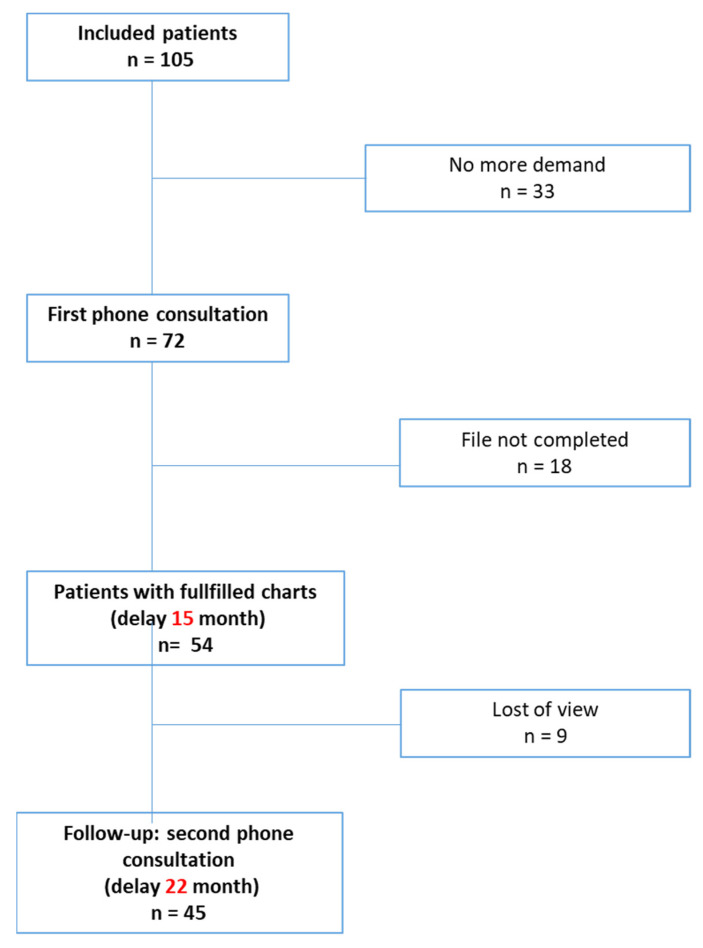
Flow-chart of the study.

As shown in Figure 2, 105 patients contacted the coordination team. For 33 patients, this first contact was not followed by further request and therefore, the advanced practice nurse’s phone consultation involved 72 patients. This full consultation allowed us to investigate in detail the history of the disease and the symptoms presented by the patient.

A complete record of the results arising from previous consultations and investigations was requested from the patient and her/his physician to avoid any further redundancy of examination and to guide patients’ support. A total of 54 patients fulfilled this requirement and received advice from the coordination team. Thereafter, nine patients were lost to follow-up and therefore, 45 received the same follow-up through the second phone call.

The delay since COVID-19 infection occurrence and the first and second phone calls were, respectively, 15.1 ± 7.8 and 22.6 ± 7.7 months.

### 3.2. Means of Contact of the Coordination Team

Interestingly, the attending physician referred only 27% of the patients to the post-COVID coordination team. In all, 45% of the patients directly contacted the coordination team and 19% of them contacted it via the regional support platform of doctors (RSP). We contacted the remaining 9% of patients during our medical activity since their symptoms were complex to treat and related to COVID-19.

### 3.3. Population Characteristics

To be able to compare patients’ evolution before and after coordination team-related support, we will present the characteristics of the 45 patients benefiting from the two follow-ups. The mean age of the patients was below 50 years, 62% were women and their mean body mass index was under 26 kg/m^2^. Ten patients regularly took part in a significant sport activity (jogging, football and natation) but we did not record more in detail their physical activities before SARS-CoV-2 infection. Nevertheless, all patients were working before infection, and none were hospitalized because of SARS-CoV-2-infection. Clinical characteristics and main risk factors of the population are presented in Table 1.

**Figure 3:**
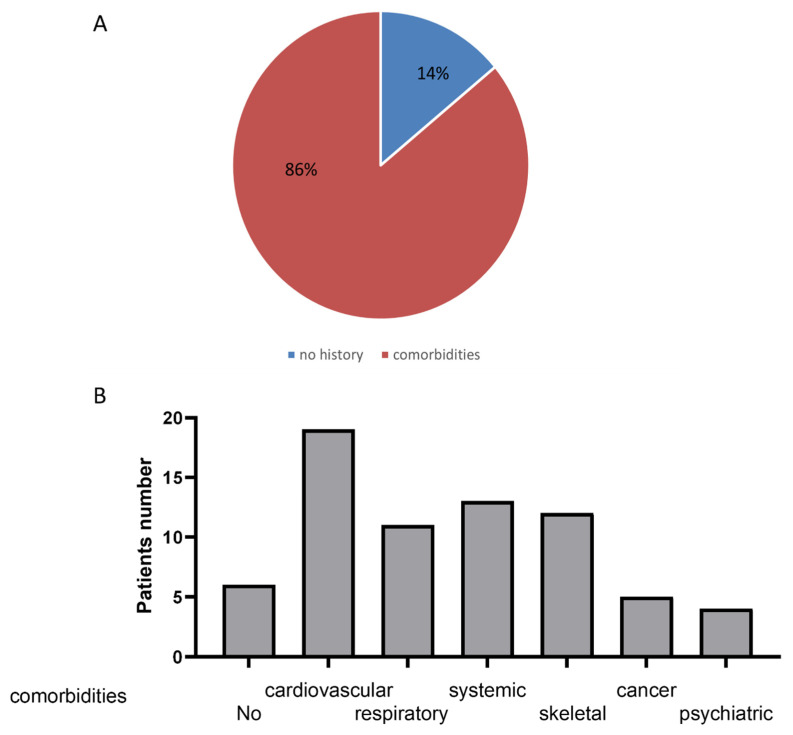
Few patients were healthy before SARS-CoV-2 infection. Proportion of healthy patients (no history) and patients with pre-existing disease (comorbidities). (**A**): Percentage of patients without comorbidities (no history) or with comorbidities. (**B**): Percentage of patients with comorbidities.

Interestingly, only six subjects (14%) were fully healthy, i.e., without comorbidity before SARS-CoV-2-infection. The most frequent pathology reported concerned the cardiovascular and the respiratory systems, musculoskeletal and systemic disorders and some patients had a history of cancer or psychiatric disease (Figure 3). 

The therapeutic proposals focused on the main symptoms reported by the patients and consisted mainly of rehabilitation. Thus, most patients had the opportunity to benefit from exercise rehabilitation (n = 24), respiratory re-education (n = 13), olfactory rehabilitation (n = 9), cognition/speech therapy (n = 4) and/or psychological support therapy (n = 11). Particularly, concerning exercise rehabilitation, fourteen patients participated in holistic care including psychological, nutritional supports and exercise such as walking, bicycling and musculation performed during 6 to 20 sessions in specialized rehabilitation centers. The other patients had mainly adapted physical activity, supervised by a physiotherapist in a primary health care facility during 6 to 22 sessions.

### 3.4. Post-COVID-19 Symptom Evolution

Fifteen months after SARS-CoV-2 infection, fatigue was the most common symptom, present in more than 90% of the post-COVID patients. Neurocognitive disorders were observed in more than 75% of cases, followed by muscle and joint pain, dyspnea and/or anxiety in more than half of the patients. Chest pain, taste and smell abnormalities and digestive problems were also present in around 40% of the patients.

As late as 22 months after SARS-CoV-2 infection, fatigue remained the most common symptom and its frequency was not decreased by the therapeutic action followed by the patients. Similarly, neurological disorders, muscle and joint pain frequency did not vary significantly. 

**Figure 4:**
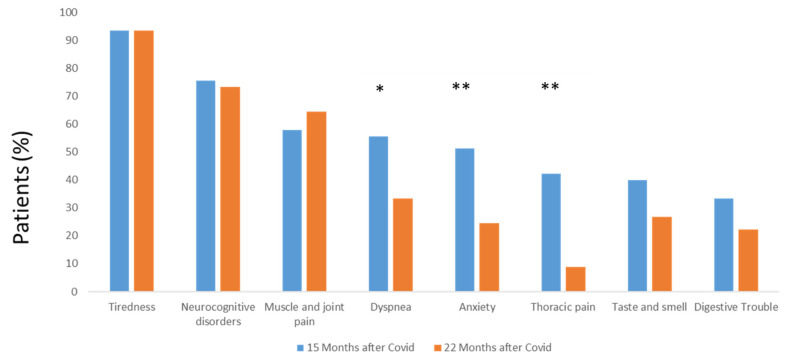
Symptoms frequency before and after coordination team-proposed support. *: *p* < 0.05, **: *p* < 0.01. Results were analyzed by McNemar’s test.

On the other hand, the frequency of chest pain demonstrated a significant reduction (*p* = 0.007, McNemar’s test), and the same result was observed concerning the frequency of anxiety and dyspnea (*p* = 0.004 and *p* = 0.013, respectively, McNemar’s test), after the coordination team support. Taste and smell and digestive trouble frequencies tended to be reduced (Figure 4).

### 3.5. Patients’ Perceived Improvement and Ability to Return to Work

**Figure 5:**
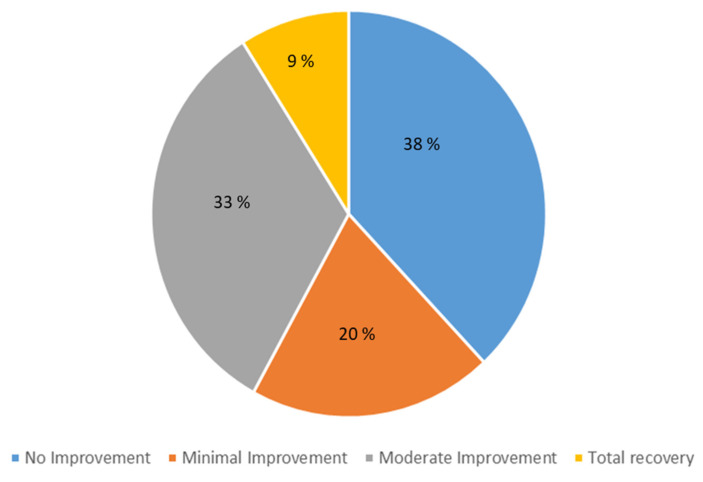
Patients’ perceived improvement.

As shown in Figure 5, only 9% of the population of complex SARS-CoV-2 patients felt healthy 22 months after virus infection; 53% of them nevertheless reported improvement, either moderate (33%) or minimal (20%). Thus, more than one-third of the patients (38%) reported no improvement in their symptoms.

**Figure 6:**
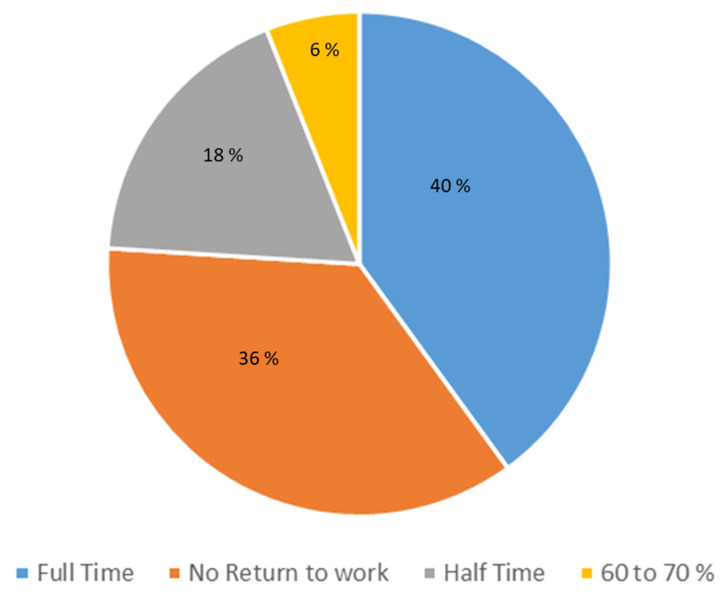
Patients’ ability to return to work.

To specify consequences of post-COVID-19 condition, we recorded the percentage of return to work. After excluding the five retired patients, we observed that 36% of the patients did not recover any work activity. However, 40% of them worked full-time and 24% worked again at least 50% of the time (Figure 6).

### 3.6. Potential Links between Patients’ Symptoms, Pre-Existing Pathologies and Back-to-Work Occurrence

**Figure 7:**
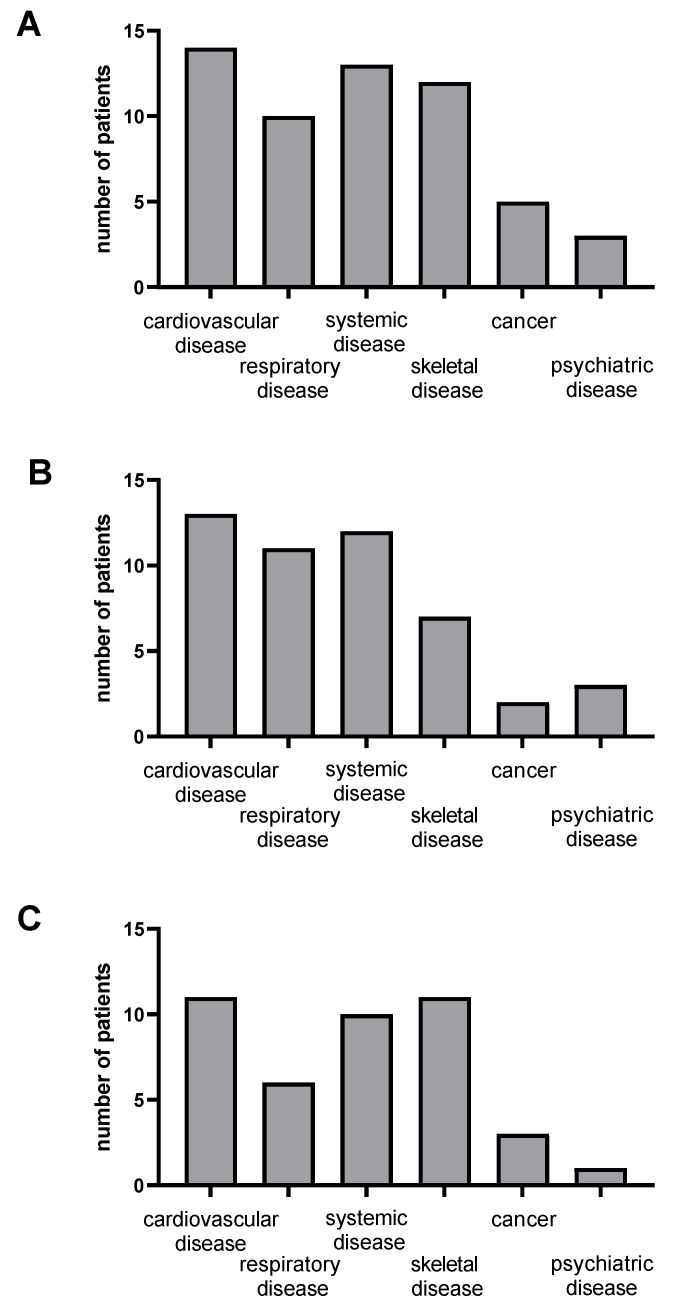
Distribution of pre-existing disease and main COVID-19-related symptoms. (**A**) Fatigue group, (**B**) neurocognitive disorders group, (**C**) pain group. No difference between groups (chi-squared test).

To investigate a possible link between their main symptoms and pre-existing comorbidities, we studied patients’ comorbidities distribution, considering the most frequent symptoms (fatigue, neurocognitive disorder and pain). As shown in Figure 7, the distribution appears balanced in these three types of symptoms; therefore, we did not observe an obvious link between pre-existing pathology and SARS-CoV-2-related symptoms. Results were analyzed by a chi-squared test, comparing the number of patients with a comorbidity (for instance, patients with previous cardiovascular disease) which was not different between the three main symptoms groups. This was also true when analyzing the other parameters.

Regarding symptoms and ability to work in the patients with comorbidities, the percentage was similar when considering the patients reporting no improvement (38%) and the patients who did not return to work (36%).

When considering specifically the six patients that were healthy before COVID-19 infection, three patients worked full-time again; one worked 50% and two did not work. Precisely, one of the latter was healthy but did not work for personal reasons. If we exclude this patient, it means that four out of five worked at least at 50% of their previous timework, which seems higher but relatively similar to observations in patients with pre-existing diseases.

## 4. Discussion

The main lessons learned from this work are that, contrary to expectations, many post-COVID-19 patients directly contacted the coordination team without going through their attending doctor. Further, the majority had significant pre-existing comorbidities; their main symptoms were those previously described, with fatigue being largely predominant. Despite the willingness to help patients and their participation in the therapeutic action proposed, with the noticeable exception of dyspnea and chest pain, which decreased significantly, self-reported patients’ symptoms often persisted and approximately one third of them were not back at work almost two years after the initial infection.

### 4.1. Post-COVID Patients Addressing and Characteristics

During this seven-month period, 105 patients contacted us directly or through their treating physicians or a medico-social structure. This number appears relatively small, but for posterity, only complex situations should have been referred, i.e., patients for whom the attending physicians could not find adequate solutions within a reasonable time. It suggests that many patients have benefitted well from the support given by their treating physician, the cornerstone of the system in France. Accordingly, the 51 patients who had not completed their file had likely obtained adapted answers to their questions upstream, without needing to fully benefit from the network set up by the coordination team.

Alternatively, although meetings with city practitioners does not seem to favor this hypothesis, this might not reflect the entire population suffering from intense post-COVID-19 condition, and one might think that all patients had no access to the coordination team. Supporting such an idea, contrary to the initial intention, treating physicians addressed the patients to the coordination team only in 27% of the cases. Thus, most patients went directly to the coordination team or through other medico-social structures. While this interpretation needs to be approached with caution, patients’ need for further medical or social support might have been more important than previously thought by their physicians.

Interestingly, the population did not totally fit with the expected characteristics of non-hospitalized patients. Although there was a majority of relatively young persons with an average age of 49 years, including a high proportion of women for whom sex was proposed to be a risk factor for several long-term post-COVID symptoms [12] and who experienced no hospitalization due to SARS-CoV-2, many of the patients were not healthy before SARS-CoV-2 infection. Additionally, the delay since COVID infection was around 15 months, suggesting that such complex situations did not resume earlier, as generally occurs. The symptoms presented by the patients had no specificity except for their very long duration [2,4,5,8,13,14,15].

### 4.2. Therapeutic Proposals and Perceived Efficacy of the Support

In all cases, with the consent of the patients, we included their referring physicians in the process, to afford that they remained the cornerstone in patient management. The precise knowledge of the attending physician of the social context of the entire family and the patient’s environment allows a better approach of everyone’s singularity. In view of the main symptoms observed and of the investigations and therapies already performed, after excluding severe diseases needing emergency support, the coordination team mainly proposed focused rehabilitation protocols. In view of the adapted rehabilitation programs, performed in specialized teams, we rather expected improvements in patients’ symptoms. Indeed, early rehabilitation is the key therapy for COVID patients [16,17,18]. Organ-related symptoms such as chest pain and dyspnea significantly decreased after re-education, suggesting therapeutic efficacy.

However, as observed during the second phone call, not all patients reported improvement, either total recovery (4%) or moderate improvement (33%). Thus, more than half of patients showed no or very little improvement (20% and 38%, respectively).

A possible explanation might be diseases pre-existing before SARS-CoV-2 infection. Thus, management of these conditions could not be optimal, resulting in patients’ symptom persistence. Indeed, many patients had relatively important comorbidities prior to SARS-CoV-2, affecting the cardiovascular, respiratory, systemic and musculoskeletal systems. Further, several patients had a history of cancer or psychiatric disorder. When talking about post-COVID-19 condition, there is a lack of data reporting reduced functions and activity in the participants compared to the previous ones, i.e., before SARS-CoV-2 infection, and further studies will be useful to investigate such issues.

We nevertheless investigated whether symptom persistence was related to pre-existing pathologies. Indeed, SARS-CoV-2 infection might modulate, or generally enhance symptoms, but as previous diseases persist, symptoms should also persist even if specific recovery from SARS-CoV-2 occurs. No clear relationship between pre-existing diseases and SARS-CoV-2-associated symptoms was observed, which is consistent with an interesting recent report supporting that comorbidities before SARS-CoV-2 did not predict persistent symptoms [19]. Further, fatigue, which was one of the more frequent symptoms, has been shown to be not related to SARS-CoV-2 severity [20]. On the other hand, we observed a tendency to a greater return to work if patients were healthy before SARS-CoV-2 infection. Unfortunately, these are few and the hypothesis that return to work is easier in patients who do not have comorbidity remains to be demonstrated. 

Alternatively, many studies are on the way to better understanding SARS-CoV-2 pathophysiology, including the potential involvement of immune, cardiac, vascular, etc., alterations [21,22,23,24] that might participate in long and complex SARS-CoV-2 symptoms and generate adequate treatment.

Finally, the authors also proposed that psychological factors might play a role in post-COVID-19 condition since the remaining symptoms (fatigue, neurocognitive disorders, pain) were very general and impacted by the patients’ subjective feelings—potentially and partially disconnected from the degree of the disease. This is largely controversial and generally badly received by the patients, who claim that they are not believed. In this view, the large reduction in anxiety we observed also suggests beneficial effects secondary to psychologic support. Thus, psychological and physical supports should not be in opposition, as both ways deserve to be used to better fight against post-COVID-19 condition. We wish to emphasize that although difficult to deal with, their feelings are a reality for the patients, and it is important for medical teams to consider them and try to improve such feelings.

### 4.3. Limitations of the Study

A larger population-based study might help to underlie the mechanisms involved and enhance strategies to treat post-COVID patients. However, the data proposed here might participate in a better knowledge of this complex situation and on its management in real life.

Self-reported symptoms are by nature subjective and validated questionnaires should add value to the results. Nevertheless, both tools likely deserve to be used and are complementary, with free answers allowing to expand patients’ feeling analysis. 

## 5. Conclusions

In conclusion, in complex situations, many patients try to refer directly to the coordination team, suggesting that the place of the attending physician should be put forward. Despite personalization of the supports, many patients (one third) felt the help insufficient and accordingly, did not return to work full-time.

However, all of them greatly appreciated to be contacted again late after SARS-CoV-2 infection, which suggest that besides the need of multi-disciplinary collaborations and early rehabilitation, including psychological help allowing for better symptom management, relatively simple actions such as a phone call might be useful to reduce patients’ feelings of abandonment.

Finally, and importantly, the best way to avoid post-COVID-19 condition is to avoid SARS-CoV-2 and, therefore, to maintain barrier gestures and to perform adapted vaccination as needed.

## Figures and Tables

**Table 1:** Population clinical characteristics and main risk factors.

		Mean ± SD	
**Clinical characteristics**	Age, years	49.6 ± 11.2	
	BMI, kg/m^2^	25.8 ± 5.6	
		**Number** n = 45	**Percentage**
	Female	28	62.2%
	Male	17	37.8%
**Main risk factors**	Hypertension	13	28.9%
	Obesity	10	22.2%
	Smoker	8	17.8%
	Hyperlipidemia	4	8.9%
	Diabetes	3	6.7%

## Data Availability

The data presented in this study are available on request from the corresponding author.
